# Consumer Satisfaction with Newborn Pulse Oximetry Screening in a Midwifery-Led Maternity Setting

**DOI:** 10.3390/ijns4040038

**Published:** 2018-12-02

**Authors:** Elza Cloete, Thomas L. Gentles, Robert A. Lutter, Kelly Richards, Kim Ward, Frank H. Bloomfield

**Affiliations:** 1 Liggins Institute, University of Auckland, Private Bag 92019, Auckland 1142, New Zealand; 2 Paediatric and Congenital Cardiac Services, Starship Children’s Hospital, Auckland 1023, New Zealand; 3 Heart Kids New Zealand, Auckland 1023, New Zealand; 4 Hawke’s Bay District Health Board, Hastings 4120, New Zealand; 5 School of Nursing, University of Auckland, Auckland 1142, New Zealand

**Keywords:** pulse oximetry, newborn, screening, informed consent, consumer participation

## Abstract

Pulse oximetry screening to detect hypoxaemia in newborn infants was introduced at birthing facilities in New Zealand during a feasibility study determining barriers and enablers to universal screening in a midwifery-led maternity system focused on community values and partnership with, and participation by, consumers. During the 2-year study period, parents of infants who underwent pulse oximetry screening were invited to complete a written survey to investigate consumer satisfaction. Respondents ranked their satisfaction with the test and with information resources on a five-level Likert scale. Additional comments were added in a free text space. Participation was voluntary and anonymous. A total of 657 surveys were included for analysis. Consumers were satisfied with the screening procedure; 94% either agreed or strongly agreed that it is an important health check. Although the quality of information sources was deemed good, a third of participants indicated a wish to obtain more information. Some participants stated that retention of information was an issue, reporting that they were fatigued following the birth. Consumers are receptive to pulse oximetry screening. Sharing information (while considering the receptivity of parents) and engaging the parents of newborn infants are factors that are paramount to the success of newborn screening initiatives.

## 1. Introduction

Pulse oximetry has been widely adopted as a screening test for the detection of critical congenital heart disease in newborn infants [1]. Different approaches have been taken to the delivery of the screening test, including ad hoc hospital-based screening [2,3] and initiatives aimed at the implementation of national or state-governed screening programmes [4,5]. New Zealand currently does not have a policy on pulse oximetry screening and is considering which approach to take. Factors specific to the national model of maternity care are important in policy development, as well as consideration of the international experience.

The New Zealand Health Strategy is based on five strategic themes: people-powered, closer to home, value and high performance, one team, and smart system [6]. Decisions on the delivery of screening programmes in New Zealand are based on five key principles, namely: safety, efficiency, effectiveness, equity, and consumer acceptability [7]. A focus on people and the extent to which a service meets the needs of consumers is therefore at the core of the decision-making process when a new health service is introduced. Incorporating community values, informed consent, and protection of patients’ rights have been highlighted as critical factors in the delivery of healthcare services in New Zealand [8].

This study investigates the consumer acceptability of pulse oximetry screening in the New Zealand healthcare setting. Furthermore, we explore the challenges associated with balancing the consumer’s right to informed consent with the potential negative effect on participation when consumers are burdened with a complex screening process.

## 2. Materials and Methods

Pulse oximetry screening to detect hypoxaemia in newborn infants was introduced as part of a research study at hospitals and primary birthing units affiliated with three District Health Boards located in New Zealand’s North Island. The study was conducted between May 2016 and April 2018. The screening test was performed by midwives or nurses after obtaining written informed consent from parents. Screeners received training and education that covered how to conduct the test, conversations with parents including obtaining informed consent, and provision of feedback on screening results. Consumer information resources, including an information brochure and video, were available throughout the study online and in print (brochure) [9].

Parents of infants who underwent pulse oximetry screening were invited to complete a survey, which was distributed and collected prior to discharge home following the birth of the child. Participation was voluntary and anonymous. Extending invitations to parents to complete a survey was at the discretion of participating centres. A written survey was designed in collaboration with consumers with the aim of investigating satisfaction with the screening test, and to determine whether information about the test was useful and disseminated effectively. The first five questions were related to participant demographics. The following eight statements were related to the screening test or the information/resources provided. Respondents were asked to rank their satisfaction with the test and information resources on a five-level Likert scale ranging from “strongly agree” to “strongly disagree”. The survey also enabled participants to state if they had not received the brochure or viewed the video. A free text space was provided where any additional comments could be added.

Quantitative data were analysed using descriptive statistics. Categorical variables are expressed as numbers and percentages, and continuous variables are presented as median and range. Free text comments were analysed thematically. Comments such as “thank you”, or “no comment” were excluded due to lack of analytic specificity. Quantitative and qualitative data were then synthesised into three main themes.

This study was approved by the Health and Disability Ethics Committees of New Zealand (15/NTA/168).

## 3. Results

During the study period there were 27,404 births in the participating regions. Of these, 16,644 (61%) infants were screened. Six hundred and fifty-seven surveys were completed and returned to the research team, which represents 4% of pulse oximetry screening study participants. The characteristics of survey participants and that of infants that underwent screening are summarised in Table 1. Primary and secondary birthing facilities were better represented amongst survey responders; ethnic spread was similar in survey responders and the whole cohort.

Analysis of survey results and comments revealed three themes: (1) parents were satisfied with the screening procedure; (2) the quality of the available information was good, but not all received sufficient information, and (3) the timing of information delivery influenced retention of information.

### 3.1. Theme 1: Parents Were Satisfied with the Screening Procedure

**Figure 1:**
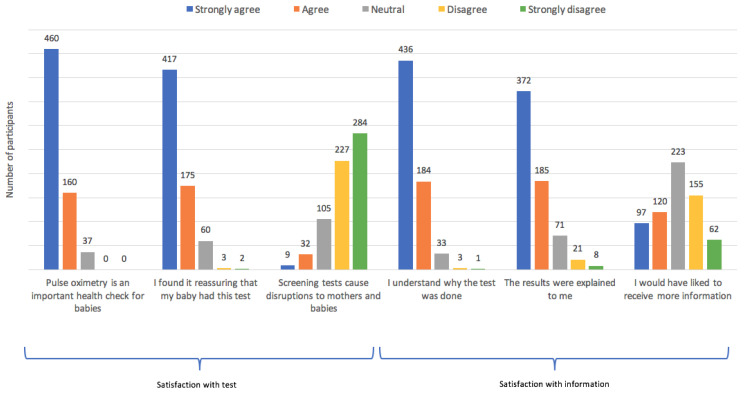
Overall consumer satisfaction.

The vast majority (94%) of parents either agreed or strongly agreed that pulse oximetry is an important health check for newborns and 90% found it reassuring that their child had the screening test. Only a small number of participants (6%) expressed that screening tests caused some disruption to mothers and their newborn infants (Figure 1). Satisfaction with the test was highlighted by 24 participants who added an additional comment in writing (Table 2). Comments reflected participants’ views that the test was simple and fast, and they supported the importance of identifying issues early.

### 3.2. Theme 2: The Quality of Available Information Was Good, But Not All Received Sufficient Information

**Figure 2:**
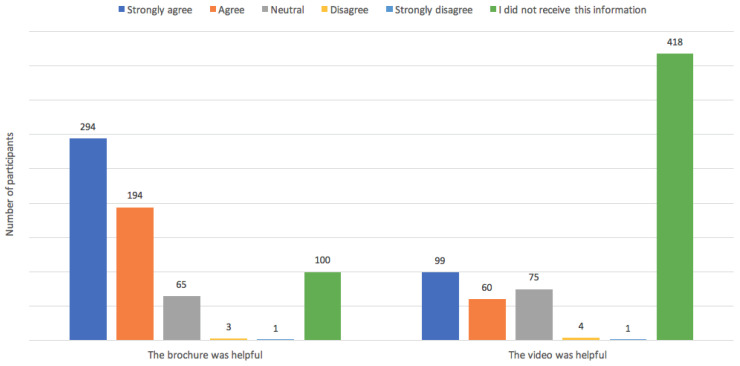
Consumer satisfaction with information resources.

Parents reported that they understand why the pulse oximetry test was offered to them and agreed that the result of the test was explained adequately. A third of participants indicated a wish for more information. The remainder were either impartial or satisfied with the quantity of information they received (Figure 1). There was positive feedback for the parent information brochure, with 74% agreeing that the content was helpful, but one hundred respondents (15%) did not receive this source of information. The parent information video was not well distributed with the majority (64%) reporting that they had not viewed it. Among the 239 who did watch the video, 159 (67%) reported that it was a useful source of information (Figure 2). Twenty participants provided additional written comments in relation to the quality and dissemination of information. Some expressed a wish for more information, particularly concerning the results of the test. Examples are provided in Table 2.

### 3.3. Theme 3: Timing Influenced the Retention of Information

Although the survey did not ask specifically about the timing of provision of information, 12 participants made a written comment addressing this topic. They described poor recollection of the test and of the information that was provided. Some indicated that they were fatigued following the birth of their child and therefore could not retain the information (Table 2).

## 4. Discussion

This study has shown that parents are receptive to a pulse oximetry screening test for hypoxaemia and it is considered to be an important health check for babies. The majority of parents were satisfied with the amount of information provided to them. However, some commented that it was difficult to retain the information when it was received shortly after the birth of their baby.

The model of parental consent, and the implications of false-positive and false-negative results, should be taken into consideration when developing goals for consumer participation in a screening programme for newborn infants. Due to the nature of our screening initiative the Health and Disability Ethics Committee required that written parental consent be obtained prior to undertaking screening. It may have impacted on the uptake of screening in the study, for which a 61% screening rate was achieved. Similar studies have reported that written consent for screening tests can be a burden to health care providers [10] and may arouse suspicion and mistrust among consumers, which can make decision-making harder [11]. Moreover, the mere provision of information can provoke anxiety by exposing parents to worrying probabilities when the risk of these is low. Some hypothesise that parental anxiety can be alleviated if pulse oximetry is “delinked” from critical congenital heart disease and should simply be performed as part of the routine documentation of a vital sign. The need for parental consent, and detailed explanations of the test and of congenital heart disease, are deemed unnecessary when this informal approach is taken [12]. This approach argues that there is no need for sharing information with parents when they are not involved in the decision-making and, therefore, the process of screening is simplified significantly. The findings of this study do not support this argument and demonstrate parents’ desire and appreciation for clear and accurate information. Manson reported that the desire for information is separable from the desire to be involved with decision-making. Consumers may want information in order to prepare themselves for a treatment, its consequences or its risks [13]. The purpose of providing information therefore extends beyond obtaining agreement from parents for the test to be performed. Sharing information promotes trust in the provider by demonstrating transparency and knowledge, as well as respect for the infants and their parents. The appreciation that parents have of being well informed with matters relating to their newborn child clearly came across in this study. Furthermore, informed parents may experience less anxiety if their baby has an abnormal screening result.

The majority of positive pulse oximetry screening results are due to diseases other than congenital heart disease [14]. However, congenital heart disease remains the primary target of oximetry screening as early identification and treatment may prevent morbidity and/or mortality in an affected infant [15]. A failed screening test necessitates a discussion with the parents during which the possibility of a cardiac defect has to be raised. In the absence of an obvious respiratory or infective cause for hypoxaemia, an echocardiogram should be performed. In New Zealand, approximately one-third of infants are born in facilities where echocardiography and/or paediatric services are not available. A positive screening result consequently necessitates referral and transfer to a larger centre. Parents who receive effective education enabling them to understand the different types of screening results and investigation pathways are psychologically better prepared in the event of a true- or false-positive result [16,17,18,19,20]. Gurian et al. reported that informed mothers experienced less stress when their newborn was subjected to repeat metabolic screening following a positive test result [16], and Clemens et al. demonstrated that parents remained very positive and supportive of their hospital’s newborn hearing screening programme despite receiving a false-positive result [19]. The false-positive rate for pulse oximetry screening has been reported to be between 0.05% and 0.5% depending on the timing of screening, with earlier screening resulting in higher false-positive rates [21]. A small percentage of parents will have this outcome and be exposed to the potential of harm. The parents of infants with positive screening results were not specifically targeted in this study, but it would be important to assess acceptability among this group in future research.

Raising awareness among parents and healthcare providers may enable the timely diagnosis of infants who receive a false-negative screening result. The information resources disseminated as part of an oximetry screening initiative should make parents aware of the fact that pulse oximetry screening will not detect all forms of cardiac disease. The signs and symptoms of cardiac disease should be discussed and parents should be encouraged to seek medical advice if they have any concerns about their baby. In our research setting, pulse oximetry screening was provided by midwives who are also responsible for the care of mothers and babies in the first 6 weeks post-partum. As part of the pulse oximetry screening initiative these midwives were provided with training and information resources that may enable them to recognise the signs and symptoms of an underlying cardiac defect. Several studies have shown that raising awareness can enable the early detection of underlying disease [22,23]. Therefore, we argue that screening processes should include information that mitigates the consequences of false-negative results.

The study did, however, find that the timing of delivering the information is an important factor for parent satisfaction. Several participants commented that they were unable to retain information that was given to them shortly after the birth of their child consistent with findings by other researchers [11,24,25]. Initiating sharing of information to raise awareness of pulse oximetry screening in the third trimester of pregnancy, in conjunction with discussions about newborn metabolic and hearing screening, vitamin K administration, and other newborn baby checks, may address this deficiency. Parental decisions are often informed by the advice they receive from their midwife and it is trust in the midwife that can lead to the acceptance of the test that is offered to them [11,25]. The role of the lead maternity carer is, therefore, likely to be central to the successful delivery of a pulse oximetry screening programme in this setting.

Consumers’ opinions on the effectiveness of information-sharing during the pulse oximetry screening study varied: a third was satisfied with the amount of information; a third impartial, and a third expressed the wish to receive more information. The structure that organised screening programmes bring can provide a platform for more effective dissemination of information. Standardising the training and education provided to those who are responsible for antenatal care and those who perform screening is more likely to result in equitable service delivery. Organised screening also yields better results than opportunistic screening in socioeconomically deprived communities, with greater participation by those with lower levels of education [26]. Equally, the importance of educating others involved with the care of newborn babies, such as paediatricians, paediatric registrars, and nurse specialists, should not be overlooked. Screeners will rely on them for advice and guidance when an infant fails to reach oxygen saturation targets. Arnold et al. reported that uninformed clinicians could undermine efforts to provide a good quality hearing screening service. Focus group discussions in that study yielded that both parents and audiologists were of the opinion that physicians need more current and accurate information to ensure that audiology services are provided in a timely manner [24].

Apart from the consideration that has to go towards consumer participation in screening programmes for infants, this research also highlighted that thought should go into the effective dissemination of information. The parent information brochure was adequately distributed and well received. In contrast to this, very few viewed the educational video and it appears that this resource added very little as an information source in this setting. The medium that was used to host this resource was perhaps not suitable for the target audience. Social media, rather than hospital web pages, may be a more effective tool in the current era.

Although this study represents a small cohort of those that participated in the pulse oximetry screening study, ethnic representation among survey participants was similar to that of the population that were screened. Over-representation of primary and secondary birthing facilities is unlikely to bias survey findings, as families in these settings are more likely to be adversely affected by positive screening results as these infants may need to be transferred for an assessment. However, in the absence of a structured recruitment method, invitations may have been preferentially extended towards parents exhibiting a positive attitude towards pulse oximetry screening. Clear themes could be identified from this survey that will aid in the design of a national screening programme for New Zealand’s unique maternity setting.

## 5. Conclusions

Screening, whether as part of a service provision or research study, requires consumer partnership and participation. This view places consumer satisfaction and information-sharing at the core of any screening initiative. Acceptability among consumers is often based on their understanding of the benefits of the test and their ability to partake in the decision-making. Therefore, sharing information and engaging with the parents of newborn infants were paramount to the success of this newborn screening initiative. Pulse oximetry screening was well received and understood by consumers in this setting and is considered to be an important health check for newborn infants.

## Figures and Tables

**Table 1:** Participant characteristics.

	Median (Range)	Screening Study, *n* (%)	Survey, *n* (%)
		16,644 (61)	657 (4)
**Age (years)**			
15–24 25–34 35–44	29 (15–44)	n/a	131 (20) 379 (58) 147 (22)
**Ethnicity**			
European Māori Pacific Peoples Other		5406 (33) 3040 (18) 2206 (13) 5992 (36)	246 (37) 148 (23) 81 (12) 182 (28)
**Number of children**			
One to three Four to six Seven to nine	2 (1–9)	n/a	600 (91) 52 (8) 5 (1)
**Highest level of educational attainment**			
Tertiary qualification Trade certificate School qualification		n/a	382 (58) 65 (10) 210 (32)
**Place of birth**			
Tertiary Hospital Secondary Hospital Primary Birthing Unit Home		13,568 (82) 1739 (10) 989 (6) 26 (2)	407 (62) 150 (23) 91 (14) 9 (1)

Abbreviations: n/a—not applicable.

**Table 2:** Sample quotes.

Theme	Example Quotes
(1) Satisfaction with screening procedure	*“No issues. Simple, fast. No complications or disruptions to me or baby.”* *“Amazing technology and such a simple test to administer. Great to have this testing available for all babies. Much better to identify potential issues early.”* *“I was very satisfied with having the screening test done and happy with the results reassuring me my baby is healthy. Thank you.”* *“Everything has been performed really well. Completely satisfied. Well done guys.”* *“I would like to thank my midwife because she did the test. It was very important for my baby.”* *“Everyone should do this. Make it mandatory.”* *“This is a vital screen—I was very glad to have it offered.”* *“Test was easy.”*
(2) Quality of information was good, but not always sufficient	“*The brochure had enough information. I think if video offered I would have said no thanks.”* *“Test was easy and I was well informed of why the screening was being done.”* *“Glad you discussed the need/reason for the test.”* *“We were given a brochure, but relied mostly on verbal information provided about the test.”* *“I was well informed of why the screening was being done.”* *“I am very satisfied because she provided all the important information that is very necessary for all parents.”*“*Don’t recall receiving any information.”* *“Need to explain more about the test to parents.”* *“Agree with doing the test, but didn’t get feedback.”* *“Lack of communication regarding results.”* *“Borderline results—not very well explained by nurse.”* *“Didn’t see a video or brochure.”*
(3) Timing influenced the retention of information	*“I don’t remember much as it was late and I was out of it. Partner explained to me we had it done.”* *“Got given brochure—did not read due to being tired. Was good to have the midwife explain instead.”* *“Would have been useful to receive the parent information in advance.”* *“The test was undertaken during our first night, so my partner and I struggled to take it all in. I am sure it was explained well at the time—just difficult to recall.”* *“Was a bit out of it when this test was performed, so can’t remember much.”* *“No memory of this.”* *“I don’t remember.”* *“We can’t recall the test very well, but have a faint recollection of looking at the brochure.”*
